# Perceptions of unmet healthcare needs: what do Punjabi and Chinese-speaking immigrants think? A qualitative study

**DOI:** 10.1186/1472-6963-10-46

**Published:** 2010-02-22

**Authors:** Emily G Marshall, Sabrina T Wong, Jeannie L Haggerty, Jean-Fréderic Levesque

**Affiliations:** 1 Department of Family Medicine, Dalhousie University, 5909 Veteran's Memorial Lane, Halifax, Nova Scotia, B3H 4H7, Canada; 2 School of Nursing and Center for Health Services and Policy Research, University of British Columbia, 2211 Wesbrook Mall, Vancouver, British Columbia, V6T-2B5, Canada; 3 Department of Family Medicine, Université de Sherbrooke, 1111 Rue Saint-Charles Ouest, Longueuil, Quebec, J4K 5G4, Canada; 4 Department of Community Health Sciences, Université de Sherbrooke; 1111 Rue Saint-Charles Ouest, Longueuil, Quebec, J4K 5G4, Canada; 5 Institut national de santé publique du Québec, 945 Avenue Wolfe, Québec, G1V 5B3, Canada; 6 Centre de recherche du Centre hospitalier de l'Université de Montréal, 2900, boulevard Édouard-Montpetit, Montréal, Québec, H3T 1J4, Canada

## Abstract

**Background:**

Unmet healthcare needs - the difference between healthcare services deemed necessary to deal with a particular health problem and the actual services received - is commonly measured by the question, "During the past 12 months, was there ever a time when you felt that you needed healthcare, but you didn't receive it?" In 2003, unmet needs were reported by 10% of immigrants in Canada, yet, little is known specifically about Chinese- or Punjabi-speaking immigrants' perceptions and reporting of unmet needs. Our study examined: 1) How are unmet healthcare needs conceptualized among Chinese- and Punjabi-speaking immigrants? 2) Are their primary healthcare experiences related to their unmet healthcare needs?

**Methods:**

Twelve focus groups (6 Chinese, 6 Punjabi; n = 78) were conducted in Chinese or Punjabi and socio-demographic and health data were collected. Thematic analysis of focus group data examined the perceptions of unmet needs and any relationship to primary healthcare experiences.

**Results:**

Our analysis revealed two overarching themes: 1) defining an unmet healthcare need and 2) identifying an unmet need. Participants had unmet healthcare needs in relation to barriers to accessing care, their lack of health system literacy, and when the health system was less responsive than their expectations.

**Conclusions:**

Asking whether someone ever had a time when they needed healthcare but did not receive it can either underestimate or overestimate unmet need. Measuring unmet need using single items is likely insufficient since more detail in a revised set of questions could begin to clarify whether the reporting of an unmet need was based on an expectation or a clinical need. Who defines what an unmet healthcare need is depends on the context (insured versus uninsured health services, experience in two or more healthcare systems versus experience in one healthcare system) and who is defining it (provider, patient, insurer).

## Background

An integral objective of healthcare systems, especially the primary healthcare sector, is to respond to peoples' perceived need for care. With enhanced access to care and improvements in quality of care, we would expect to see a corresponding decrease in unmet healthcare needs. Indeed, unmet healthcare need has been identified as a critical indicator of access to care within a healthcare system [1-3] and is used to compare access across different healthcare systems [4].

Unmet healthcare need has been defined as the "difference between healthcare services deemed necessary to deal with a particular health problem and the actual services received" [1]. In many population-based, national surveys (e.g., Canadian Community Health Survey-CCHS, National Health Interview Survey), unmet need is measured by a question such as, During the past 12 months, was there ever a time when you felt that you needed healthcare, but you didn't receive it? Differences in unmet needs among different population groups could represent either true differences in access to healthcare or differences in interpretation by respondents who speak a different language or have different cultural backgrounds. There remains a paucity of work that examines the conceptual, operational, or psychometric equivalence of items currently measuring unmet health needs in languages other than English. Instruments, and specific items, developed to measure constructs important to the general public may not have conceptual equivalence [5-9] (e.g., no adequate reflection of unmet needs important to groups who speak little or no English), operational equivalence [5,6,9,10] (e.g., appropriate survey methods including reading level, item format, and instructions), or psychometric equivalence [5,6,10,11] (e.g., comparable psychometric properties including reliability and validity) in groups who speak English as a second language (ESL). Moreover, without examining the concept of unmet healthcare needs in ESL groups, self-report measures of unmet needs may be inadequate.

In Canada, immigrants are an increasing share of the total population (18.4%) and constitute an increasingly important segment of Canadian society; roughly 58% of immigrants come from Asia [12]. A majority of immigrants (70.2%) in 2006 reported a mother tongue other than the official languages of English or French [13]. The largest proportion of first languages spoken in the home in childhood, among all immigrants, were Chinese (18.6%), followed by Italian (6.6%), Punjabi (5.9%), Spanish (5.8%), German (5.4%), Tagalog (4.8%), and Arabic (4.7%) [14]. In the Unites States (US), the 2003 Census reported that 11.7% of the US population, or 33.5 million people, were immigrants; one-quarter of immigrants were from Asia, over 50% from Latin America, and 13.7% from Europe [15]. British Columbia is home to the second largest immigrant population in Canada, where 50% of BC immigrants are from Asia and 30% speak Cantonese, 25% speak Punjabi, and10% are not fluent in English [16]. Recent population-based studies in Canada identified that recent immigrants reported more unmet needs for care compared to those that immigrated to Canada more than ten years ago or to people born in Canada [17,18].

Currently, there is no standardized translation of self-reported unmet healthcare needs questions in either Chinese or Punjabi. Thus, this indicator of access may be of limited use with these ethnic and mostly immigrant groups. In this paper, we describe work that examines: 1) the conceptualization of unmet healthcare needs among Chinese- and Punjabi-speaking immigrants, and 2) if any primary healthcare experiences are related to their unmet healthcare needs.

## Methods

As part of a larger study examining Chinese- and Punjabi-speaking people's primary healthcare experiences, 12 focus groups, each consisting of six to nine participants, were conducted in Chinese (n = 6) or Punjabi (n = 6). Participants were recruited through community organizations and Gudwaras (Sikh place of worship, referred to as a "Sikh temple"). Leaders of the organizations helped us identify and help recruit potential participants. Focus groups were conducted separately by language (Cantonese, Mandarin, Punjabi) in different locations across the Vancouver's lower mainland in British Columbia (BC). Given that healthcare needs and use of care are known to vary, focus groups were conducted by different age groups (< 50 years, ≥ 50 years) and gender (men, women). Eligibility criteria included: Cantonese- or Mandarin-speaking Chinese and Punjabi-speaking South Asian immigrants, aged 19-90, who had visited their primary healthcare provider at least twice in the past two years. We chose Chinese- and Punjabi-speaking immigrants because they represent the largest group of people who speak English as a second language in BC. Upon obtaining informed consent, participants filled out a short survey, in Chinese or Punjabi, about their socio-demographic information, self-reported health, and their recent experiences in using the primary healthcare system, including having any unmet healthcare needs in the past 12 months. All focus groups were taped and each participant received $15 in appreciation for their time. All procedures were approved by the University of British Columbia's Institutional Review board. Each community organization was given $75 in appreciation for their help in the recruitment process.

Focus groups were conducted by two trained bilingual and bicultural facilitators (English and Punjabi/Mandarin\Cantonese). Open-ended questions were asked about people's experiences in accessing and using primary healthcare. As part of the interview schedule, questions related to unmet healthcare needs were developed by the research team in consultation with key informant members of the Chinese and Punjabi-speaking communities. Examples of questions include: "What is the first thing you do after deciding that you need healthcare or advice?"; "What are the most important things to take into consideration in choosing this specific provider/place to go for healthcare or advice?"; "During the past 12 months, was there ever a time when you felt that you needed healthcare, but you didn't receive it?" This paper reports on a secondary analysis conducted to examine participants' discussions of unmet healthcare needs within the context of primary healthcare.

Data were transcribed and translated into English by one of the facilitators. Five percent of the English transcripts were randomly chosen to check against the taped focus group, by a different bilingual and bicultural research assistant, for accuracy of words and concepts being discussed in the translation. We found less accuracy in the English translation of the Chinese focus groups since there were two dialects of Chinese, Cantonese and Mandarin, used for facilitating the focus groups. Therefore, each Chinese focus group tape and English transcript was double-checked by a bilingual, Cantonese or Mandarin and English-speaking research assistant in their entirety. Once the English-equivalent data were cleaned, we organized it into codes based on an agreed upon coding scheme. All transcripts were coded by at least two members of the team. A qualitative software program, ATLAS.TI, was used to organize the coded data across all participants.

We used thematic analysis and a phenomenologic approach for our methodological framework. Phenomenology is a philosophy and a method through which we examined participants' primary healthcare experiences and how these experiences related to the phenomenon of unmet healthcare need [19]. Initial data analysis involved immersion in the data as a whole [20], so transcripts of focus groups were reviewed by all members of the team in their entirety and discussed. Meaning of sentences and experiences were considered in relation to the complete transcript. Then a disciplined and systematic search was used to identify themes [20]. Thematic analysis, a method for identifying, analyzing and reporting patterns or themes within data [21] was used to illuminate experiences of unmet health care needs.

## Results

Seventy-eight people participated in one of the twelve focus groups. Focus group participant's characteristics are shown in Table 1. For descriptive purposes, the data are shown by language and age group, as well as the total. Most participants (83%) were married, had a diploma or degree (51%), and 42% reported having a household income between $20,000 and $49,999; one-third were employed full-time (Table 1). Chinese participants were less likely than Punjabi counterparts to have chronic conditions (41% vs. 73%) and more likely to report being in excellent, very good, or good health (79% vs. 69%).

**Table 1:** Chinese and Punjabi Focus Group Participant Characteristics

Characteristic	Language		Age Groups		Total (n = 78)
	Punjabi (n = 45)	Chinese (n = 33)	< 50 yrs (n = 39)	50 + yrs (n = 39)	
Gender (%)					
Male	46	54	49	51	50
Marital Status (%)					
Married/Living with a Partner	84	83	82	85	83
Education (%)					
Less than grade 12	32	7	8	31	19
Grade 12 o GED	5	20	0	26	13
Some post-Sec/College	16	17	28	5	17
Diploma/Degree	46	56	64	39	51
Income (%)					
< $20,000	28	23	7	45	26
$20,000 - $49,999	36	50	51	32	42
$50,000 - $79,999	33	23	37	19	29
$80,000 and above	3	3	3	3	3
Work Status (%)					
Full-time	35	32	54	13	33
Part-time	14	5	10	8	9
Not employed (student, retired, disability, homemaker)	46	61	31	77	54
Other	5	2	5	3	4
Language*					
English-language speaking abilities(1-4)					
Mean (SD)	2.73 (1.12)	2.50 (0.62)	2.97 (0.73)	2.24 (0.96)	2.62 (0.92)
Has a Regular provider (%)					
Yes	92	98	92	97	95
Doctor speaks native language (%)					
Yes	74	81	84	71	78
Don't know	26	3	13	14	13
Health Status (%)					
Excellent/Very good/Good	69	79	87	59	73
Fair/Poor	31	21	13	41	27
Chronic Condition (%)					
No chronic condition	27	59	67	21	44
1 chronic condition	30	34	28	36	32
2 chronic condition	27	2	3	26	14
3 and more	16	5	3	18	10

*Note. Response categories were: very well, well, not well, not at all. A higher score = more of the concept.

Almost all participants (95%) reported having a regular primary healthcare doctor, three-quarters see a doctor who speaks their native language and all participants reported being only somewhat proficient in English. Thirty-two people had been in Canada for less than 10 years and only five people reported on the survey having any unmet healthcare needs over the previous 12 months (data not shown).

Thematic analysis revealed two overarching themes: 1) defining an unmet healthcare need and 2) identifying an unmet need. Participants had unmet healthcare needs in relation to barriers to accessing care, their lack of health system literacy, and when the health system was less responsive than their expectations.

### Defining an Unmet healthcare need

Even though only five participants reported having an unmet need on the short questionnaire, more than one-third of focus group participants expressed an unmet need during the discussion. Defining unmet healthcare needs was complex for participants. While some participants were confident they had no unmet healthcare needs, others raised the question of who should identify whether an unmet need has occurred. In particular, participants wrestled with the notion that an unmet need for healthcare could be defined as not having access to existing or offered services, as well as not having access to services which are not covered by insurance benefits or publicly-funded systems. This aspect of unmet needs relates to definitions that people make of what constitutes the services one is expecting to be offered and highlights the fact that people coming from diverse cultural and socioeconomic backgrounds also have very diverse previous experiences and perceptions about the range and types of services that they expected to be available. This can impact on the declaration of unmet needs for care. For example, many participants talked about services they thought they needed but were not available through the Canadian healthcare system:

"Every time when I go back to China, I must do a whole body check-up, because you can't have it here. They don't allow you to do that, except [when] you really have a problem." (Mandarin-speaking woman, < 50 years)

Speaking only Chinese or Punjabi, in part, compounds their understanding and reporting of unmet healthcare need. As two Chinese-speaking women point out, not knowing what primary healthcare services are available or the meanings of medical terminology can affect whether they report unmet needs:

"It would be convenient [having the written information available in Chinese/Punjabi], we could understand more because sometimes when you read them [health-related brochures] in English, you really don't know what they mean, particularly the medical information. The medical terminology is very hard, such as the terms of diseases or [medical] treatments. You have to look them up in the dictionary. Sometimes we just don't bother and end without knowing the meanings." (Mandarin-speaking women, < 50 years)

"Language is a very big barrier for us Chinese. Plus, we are not doctors, of course we don't know those medical terminologies. So we really don't know what benefits that the healthcare system provides to us. We hear of some here or some there. It's not complete. It would be helpful if we are provided the information in Chinese that systematically tells us the services we can enjoy and those we are not entitled. Since the information we hear from others are not in details and not official." (Cantonese-speaking woman, < 50 years)

### Identifying Unmet healthcare needs?

Participants' discussions brought into question what should be considered an unmet healthcare needs within the context of primary healthcare. Some participants reported foregoing certain healthcare services, such as dental care and speech therapy services because they were too expensive. The acceptability of paying for certain services influences people's identification of an unmet need for care. When people receive services (the need has been met) but have to pay out-of-pocket, unmet needs are identified based on what they expect from the health service delivery system. Participants made tradeoffs between an unmet need and paying out-of-pocket for health services:

"I have seen that there is a great demand for a speech therapist, but because they are so expensive, most people do not go to them. They take $75 for each appointment; most people do not go, as it is very expensive." (Punjabi-speaking woman, < 50 years)

"He/she (the dentist) said that you have two teeth with cavities, and you have gingivitis as well. I'll make a treatment plan for you. The total will be more than two thousand [dollars], almost three thousand. The dentist talked about the medical insurance and said I need to pay half of the price. But still it's very expensive, so I gave up the plan." (Mandarin-speaking woman, < 50 years)

"I feel many times I can't get what I request. For example, I don't have diabetes, but I want to check. But the basic MSP [medical services plan] does not cover this. So even if I'm willing to pay for the test, they still can't accept me." (Cantonese-speaking man, ≥ 50 years) Some experiences with the primary healthcare system (PHC) were closely linked with discussions of unmet needs, including: accessibility, health system literacy, and responsiveness of the PHC system.

#### Accessibility

Participants discussed unmet healthcare needs as related to a variety of dimensions of accessibility including: a lack of choice in the gender of a provider, particularly for women preferring to have a female physician or a lack of a primary healthcare provider who was accepting new patients and speaks the patient's language. This highlights that their preferences for what constitutes an appropriate response to one's needs could also determine the occurrence of unmet needs. In some cases, women chose to forego cervical cancer screening because they did not feel comfortable with their male doctor providing this service:

"I haven't done my gynaecological screenings for a couple of years, because he's a male doctor. I've never requested him to do the gynaecological screening for me. I always do the screening when I go back to China and I pay for it." (Mandarin-speaking woman, < 50 years)

"I had a problem, I went to a doctor, he had to see me from above my waist......my body, my daughter had gone with me, I said to my daughter that if you will get me checked by a lady doctor, then you take me in, otherwise no. And then the doctor came, and I said in front of him, that I will not show myself to him at all." (Punjabi-speaking woman, < 50 years)

Another dimension of accessibility is seeing a doctor who is bilingual in English and Chinese or Punjabi. Participants likely have language barriers because many could not read or write in English for the purpose of a medical visit and that they would wait to see their doctor, who spoke their language. Indeed, they experienced wait times in order to see their Chinese- or Punjabi-speaking doctor:

"The time when you need care, if you don't get the appointment at that time, then you can't go to the doctor. The doctor says come after 3 days or 4 days. I don't have time to make an appointment [3 or 4 days later]. This is a difficulty." (Punjabi-speaking man, ≥ 50 years)

#### Health system literacy

Knowing how to use the primary healthcare system and what resources are freely available was also discussed in relation to unmet healthcare need. Lack of knowledge about health systems organizations and services related to the immigration process can also impact on unmet needs for care. Some had limited knowledge on how to get needed healthcare services and what services are available to them (particularly those covered under the universal healthcare plan):

"They (patients) should know which medicines are free for them, which services are free and till what age. These things should be told to new immigrants, because they do not know these things, and it is hard for them when they come all by themselves [to another country] with their families. There should be some classes and workshops so that they can get information." (Punjabi-speaking woman, < 50 years)

"I think doctors, particularly for us new immigrants, like what they mentioned, since we didn't grow up here, the family doctors, now that they are called "family" doctors, they should tell us when we first see them which services they can offer us. Otherwise we won't know which problems we should seek help from them, doctors or nurses. They should tell us which problems, for example, when you have mental problems, you should see...It is the first time for me to know that you should see your family doctor for it. I don't know it. I don't know when I should go to see him (family doctor) and when I shouldn't. I don't know in which circumstances I could be referred to a specialist. I don't know these at all." (Mandarin-speaking woman, < 50)

All participants were immigrants who were most familiar with using their home country's (India or China) health system. Less familiarity with the Canadian healthcare system (e.g., primary healthcare is typically the first contact with the healthcare system) was a source of concern for participants. Differing expectations for health service delivery was discussed in the context of not being able to obtain "immediate care" and having to "wait" for care:

"We know if we went to the hospital (in China), we went to the emergency room, we would be treated immediately by vein injection, dropping or whatever. They would treat you in a way that you could see the immediate effects, so you would have a peace of mind. But here, it takes time no matter whether you have to experience the emotional distress. You have to take Tylenol or whatever first for three days, even though sometimes you know very well that Tylenol won't help. That makes you worried because you know Tylenol won't work, so why don't you control the problem with a method that works in the first place?" (Mandarin-speaking woman, < 50 years)

#### Responsiveness

Some of the structural constraints on how primary healthcare is delivered in a fee-for-service (FFS) model created more risks for unmet healthcare need. Despite language concordance between the family doctor and participants, there was discussion across the focus groups on how the system was not responsive to their needs because of limited consultation time with the family doctor. In the FFS system, primary healthcare visits have been limited to a maximum of 15 minutes with the time per visit per patient varying based on the provider's assessment of the health issue:

"The doctor was very bad because when we went to see her, she gave us a very short time. For example, she would stop us if we wanted to ask her a tiny bit more question. Can't ask. When the reports of the lab tests came, and when we wanted to know a bit more about why the results were like that, she didn't want to answer." (Mandarin-speaking woman, < 50 years)

Moreover, there are no standard guidelines or best practices that define how a provider should be responsive to patients:

"I have left so many messages for the family doctor, but she has not called me back even once till now. I wanted to get some further information on how I can get home support. We are tax payers and we hear that home support people come and take care of the old people, but I don't know who can arrange this for us." (Punjabi-speaking woman, < 50 years)

Although most participants had a regular provider who could speak their native language (Chinese or Punjabi), participants discussed having unmet needs outside of regular office hours with other doctors:

"In India, if we see the system, whenever we need any facility, whenever we need the doctor, we can go to him at midnight also. There is 24 hour service there. But here we don't get that service. Here there are only very limited hours of service. On Saturdays and Sundays we can't get the [Punjabi-speaking] doctors, but in India the doctor is available to us all the time." (Punjabi-speaking man, ≥ 50 years)

## Discussion

Asking patients about unmet healthcare needs is complex. The analysis presented here suggests two issues in need of further consideration. First, measuring unmet healthcare needs using single items is likely insufficient since more detail in a revised set of questions could begin to clarify whether the reporting of an unmet need was based on an expectation or a clinical need. Simply asking whether someone ever had a time when they needed healthcare but did not receive it could underestimate unmet need. These results suggest that who defines what an unmet healthcare need is depends on the context (insured versus uninsured health services) and who is defining it (provider, patient, insurer). Using the current unmet needs question, "During the past 12 months, was there ever a time when you felt that you needed healthcare, but you didn't receive it?" [14] may not, by itself, be a valid indicator of unmet need. Given that large surveys may only have space for a single question about the presence of unmet healthcare needs, one alternative would be to conduct analyses that take into account language spoken at home, recency of immigration, and ethnicity. Another alternative would be to use an index reflecting different types of health needs, which may help people differentiate between their expectations and their health needs. A multidimensional measure could provide more information on the types of health needs that can be met by health plans. For example, Katz and colleagues [22] measured different types of unmet needs. Unmet need for home healthcare was defined as needing the service in the previous six months but not having received help at home (with medical problems, personal care, house-keeping, or other services) during the same period. Unmet need for emotional counselling was defined as needing the service in the previous 6 months but not having seen a mental health provider, attended a support group, or seen a spiritual care provider (e.g., minister) during the same period [22].

This study adds value to what is known about measuring unmet needs. How unmet healthcare needs is conceptualized and who defines if the need is unmet (e.g., patient, provider, parent) warrants further investigation. Chinese- and Punjabi-speaking participants, in particular, have challenges in understanding what unmet healthcare needs are or how they should be defined. Asking for a self-report of unmet healthcare need requires more explanation of what should be considered an unmet need and whether self-report methods are complementary to other methods in measuring unmet need such as provider reported unmet needs of patients or whether people received services based on clinical guidelines. In addition, collecting information about what people expect from the publicly insured healthcare system and what is considered uninsured services could help in understanding the impact that expectations might have on the reporting of unmet needs for care. Patients often have specific expectations of their healthcare visit and of the healthcare system [23]. Moreover, more work needs to be done in developing reliable and valid measures that can examine the strength of the relationships between unmet need, individuals' expectations of their health visits, and utilization of primary healthcare.

Second, experiences with a different healthcare system can create different expectations for the types of healthcare services available and how people can access these services. In British Columbia (BC), Canada, primary healthcare provides a range of services and is the place of first contact with the healthcare system for most people. In other countries such as China, primary healthcare is not necessarily the place of first contact care. People can directly access specialists. Moreover, all participants were immigrants who had varying degrees of familiarity with the BC health system. What was once a service available in their country of origin (e.g., full body check) was not available in BC and therefore, had become an unmet healthcare need. Clearly, being able to identify what healthcare service is needed affects whether people will report an unmet need.

Self-reporting of unmet healthcare needs is dependent on types of services (e.g., insured versus uninsured services), individual preferences, responsiveness of the healthcare system (e.g., hours of operation), and an individual's characteristics (e.g., language) [24,25]. Notably, people who are willing to pay out-of-pocket for uninsured services may not report unmet needs if they can obtain the service privately or in a different country such as China or India.

These insights should be considered in the light of the study limitations. As with single methodological approaches using a qualitative approach, the goal is to reach representative credibility [26]. While these results are internally valid to this study, triangulation of using mixed-method approaches are needed to enhance our knowledge about how to measure unmet healthcare needs across groups who speak English as a second language. Notably, the use of two distinct cultural and linguistic groups allowed us to identify themes applicable to both groups. Using focus groups may not have allowed for the depth of discussion that occurs with individual interviews. However, interactions with other participants can trigger memories and broadens the scope of experience that can be explored. A quantitative psychometric analysis could begin teasing apart whether the single unmet healthcare need question works differently across language groups or ethnicity. Finally, we examined the concept of unmet healthcare needs among those people who had access and had visited their primary healthcare provider at least two times in the past two years. More work is needed in understanding unmet health care needs from those who do not have a usual source of care or have not accessed primary healthcare services recently.

## Conclusions

Despite these limitations, this is the first report that we know of to examine unmet healthcare needs in Chinese- and Punjabi-speaking immigrants and it provides information on how we can move forward in more clearly defining what is meant by unmet healthcare needs in primary healthcare. These results suggest that the concept of unmet healthcare needs is multidimensional depending on who is defining what healthcare need has gone unmet [27], and recall bias [28]. Self-reported levels of unmet healthcare need are likely to elicit the most important or most bothersome memories. Therefore, using a single question should be considered a partial assessment of unmet healthcare needs. More work is needed to examine the extent to which a single question adequately captures unmet healthcare needs since it could either underestimate or overestimate the proportion of respondents who experience difficulty obtaining needed services. Unmet healthcare need, defined from a patients' view is a partial reflection one's ability to identify a health need, access the appropriate services, expectations of available services, and prior experiences using the healthcare system. Finally, English-language ability and immigration and use of a different healthcare system appear to be factors that influence the reporting of unmet healthcare needs within the context of primary healthcare.

## Competing interests

The authors declare that they have no competing interests.

## Authors' contributions

EM assisted in the acquisition of data and jointly led analysis and interpretation of the data. SW conceived of the study and design, oversaw all aspects of data acquisition and led analysis and interpretation of the data. JH and J-FL contributed to the design of the study and were involved in the analysis of data. All authors have been involved in drafting and critically revising the manuscript. All authors have given final approval of this manuscript.

## Pre-publication history

The pre-publication history for this paper can be accessed here:

http://www.biomedcentral.com/1472-6963/10/46/prepub
